# Commercial Formulation of Chlorpyrifos Alters Neurological Behaviors and Fertility

**DOI:** 10.3390/biology9030049

**Published:** 2020-03-07

**Authors:** Enoka P. Kudavidanage, D. M. I. Dissanayake, W. L. Rangi Keerthirathna, N. Lasni Wathima Nishshanke, L. Dinithi C. Peiris

**Affiliations:** 1 Department of Natural Sciences, Sabaragamuwa University, Belhiloya 70140, Sri Lanka; enoka@appsc.sab.ac.lk; 2 Department of Zoology, Faculty of Applied Sciences (Center for Biotechnology), University of Sri Jayewardenepura, Nugegoda 10250, Sri Lanka; indeewarie92@gmail.com (D.M.I.D.); rangee9183@gmail.com (W.L.R.K.); lasni.nishshanka@gmail.com (N.L.W.N.)

**Keywords:** Judo 40, chemical formulation, chlorpyrifos, organophosphate, sexual behavior, pain perception, fertility index

## Abstract

Pesticides are known to result in toxic insult. We aimed to evaluate Judo 40, the commercial formulation of chlorpyrifos on the neurological activities, fertility, and hormone levels of male rats. Male Wistar rats were treated orally with 1 mL of 20 or 50 mg/kg Judo 40. The doses were administered four times, twice a day. Sexual and exploratory behavior indices, fertility indices, serum androgen levels, blood acetylcholinesterase (BChE) levels, and neurological and muscular effects were evaluated. Serum testosterone and luteinizing hormone were significantly reduced in the rats receiving 50 mg/kg Judo 40. A reduction in viable implantation sites and live pups born were evident in the female rats mated with the male rats treated with the highest dose. Similarly, in the rats treated with the highest dose of Judo 40, a significant reduction in plasma BChE enzyme was observed. According to the results, prolonged Judo 40 exposure can cause impairment of the neurological alterations and sex hormones leading to impaired fertility. Therefore, chemical handlers should be educated on protection and risk minimization.

## 1. Introduction

The use of organophosphorus pesticides (OPs) has increased dramatically to meet the food demands of the growing population. Organophosphates are potentially hazardous to individuals involved in manufacturing, formulation, and application [1]. OPs are known for their wide application in industries, agriculture, and public health [2]. Most of the available literature links the disruption of the reproductive success of animals [3,4,5,6,7] including humans [8,9], to the neurotoxicity of pesticides [7]. OPs are derived from esters, amides, or thiols, and their toxicity could be attributed to the interruption of nervous conduction by the inhibition of acetylcholinesterase (AChE), plasma and hepatic carboxylesterase, and other esterases [10,11]. Recent evidence suggests that prolonged exposure to chlorpyrifos (CPF) can be detrimental to nontarget organisms [4,12] including humans [11,13]

Among the pesticides imported to Sri Lanka, chemical formulations comprising chlorpyrifos (*O*,*O*-diethyl-*O* (3,5,6-Trichloro-2-pyridinyl) phosphorothioate) formed more than 50% of all organophosphate insecticides [12]. In early 2000, the sale of CPF was banned by the Environmental Protection Agency in the United States. However, since farmers in the US rely heavily on this insecticide, the Agency decided to reverse the provision in 2017 [13]. In Sri Lanka, farmers apply CPFs intensely to minimize pest damage to coconut, rice, and vegetables [14]. When released to the environment, chemical formulations of CPFs such as Judo 40 will undergo evaporation, hydrolysis, oxidation, photolysis, and microbial metabolism [15]. Owing to active soil absorption and the low water dissolubility of CPFs, this can result in the contamination of percolated water, runoff water, and sediments [16], leading to a high accumulation of CPF residues, thus posing acute and chronic health risks to consumers [17]. 

It has been documented that the occupational exposure of humans to pesticides, including CPFs, are high [18]. However, in developing countries, small-scale farmers are continuously exposed to pesticides through mixing, filling, and spraying using backpack sprinklers [19]. They are further at a high risk due to the limited usage of protective gear and lack of awareness on safety [20]. In Sri Lanka, farmers are inclined to use about 30%–40% higher quantities of chemical formulations of CPF than the dose levels recommended by the manufacturers [21]. Growing evidence also indicates that CPF exposure can result in fetal developmental problems, including issues with the weight and head circumference of newborn babies [22], and high incidences of prostate and lung cancers in adults [23]. Alvarez et al. [24] reported that the soil can recover up to 25 ppm of CPF and the percentage decrease with increasing of the initial concentration [25]. The use of commercial formulations of pesticides can aggravate the toxic effects more than use of the pure active ingredient [26]. 

The active ingredient of Judo 40, CPF, is principally responsible for the accumulation of acetylcholine (ACh) as a result of inhibition of acetylcholinesterase (AChE) action in animal tissues, and is highly toxic to many animals including copepods, amphipods [27], fish [28], birds, and mammals [29]. It was reported that chronic exposure to 30 days of chronic doses (5 and 10 mg/kg) of CPF led to a decrease in sperm motility and count though a reduction in testosterone hormone and increasing amounts of the follicular-stimulating hormone [30]. It has been shown that exposure to toxicants could significantly decrease memory [31], behavior [32], and pain perception [33]. Similarly, sexual behavior is important for successful fertilization. Sexual behavior takes place via the neuro–gonadotropin axis and, therefore, any alterations to nerve transmission can lead to impairment of sexual behavior and thus fertility.

When animals are in a unique environment, exploratory behavior becomes an important survival strategy. Exploratory behavior enables animals to become familiarized with the environment, mark their territory, discover the most cost-effective utilization of available resources, and avoid predators [34]. Although exploration is vital for gathering useful information, the risk of predation is very high. Exploratory behavior depends upon the balance between the potential gain of resources and the risk taken [35]. Furthermore, exploratory behavior is a useful tool to determine drug-induced central nervous system effects. Since people of developing countries, like Sri Lanka, use minimum protective gear when spraying pesticides [14], sub chronic and chronic exposure to chlorpyrifos could result in behavioral and neural alterations in children, adults, and animals [36]. 

Aside from its neurotoxic and developmental effects, CPF is also known to adversely affect sperm chromatin condensation and sperm DNA integrity [37], the androgen profile, and fertilizing ability [30]. Previously, we showed that Judo 40, a widely applied commercial formulation of CPF, can impair the sperm motility and nuclear integrity of rats without altering the cauda epididymal spermatozoa number [11]. 

Previously, we showed that Judo 40 can impair sperm motility, to induce genotoxicity [11]. Considering the ability of Judo 40 to remain in the environment, and its extensive use and the limited literature on it, here we used matured male Wistar rats as a laboratory model for humans to study the direct effects of Judo 40 on pain perception, exploratory behavior, sexual behavior, and fertility.

## 2. Materials and Methods

### 2.1. Chemicals

Chemicals were obtained from Sigma Chemical Company Ltd. (St. Louis, MO, USA). The test material Judo 40 (active ingredient, CPF, 400 mg/mL) was purchased from Lankem Ltd., Colombo, Sri Lanka. 

### 2.2. Experimental Animals

Male Wistar rats, six weeks old and weighing between 250–325 g were purchased from Medical Research Institute, Colombo, Sri Lanka. The rats were acclimatized for one week in an animal house under standard conditions (relative humidity 50% ± 5, temperature 25 ± 2 °C, 12 h day and night cycle). Animals had free access to pelleted food (Vet House Ltd., Colombo, Sri Lanka) and tap water. The experiments were performed as per committee for the purpose of the control and supervision of experiments on animal norms after obtaining the institutional animal ethics committee clearance (ethical approval No.: 25/16).

### 2.3. Experimental Design

Upon acclimatization for one week, 27 male rats were divided randomly into three groups (n = 9/group based on our preliminary study). Animals in the first two groups were gavaged with 1 mL of 20 mg/kg and 50 mg/kg Judo 40. The third group received 1 mL of corn oil (control group). The doses were administered four times, twice a day, between 08:00–09:00 and 15:00–16:00 on two alternating days. The animals were observed daily between 9:00–11:00 and between 15:00–17:00 for overt toxic signs. Sexual behavior and fertility effects were studied on posttreatment days one and seven.

### 2.4. Dose Justification

The doses (20 and 50 mg/kg) corresponded to 1/5 and >1/2 LD_50_ (Lethal Dose), respectively. The doses were established based on our previous study [11]. We conducted an initial study to determine the dose levels. We found that the no-observed effect of Judo 40 was seen at 25 mg/kg. Hence, the doses were set to 20 and 50 mg/kg (Table A1).

### 2.5. Behavioral Studies

#### 2.5.1. Exploratory Behavior

Animals were subjected to behavioral studies on posttreatment days one and seven using an open field test according to the method described previously [38] and hole-board method [39]. Briefly, a wooden box (97 cm diameter × 32.5 cm height) was used to determine open field behavior. The box was divided into three circles, which were in turn subdivided into 19 white lines. Each rat was individually positioned in the midpoint of the field and the parameters, including locomotor activity or the number of lines crossed with both feet and rearing incidences or the frequency of animals standing on their hind limbs, were determined. Before introducing each animal, the arena was cleansed with ethanol.

The hole board, which was an open arena with four central holes of 3 cm diameter and 2 cm depth, was used in a temperature-controlled room with minimum sound and dim red lighting. A single rat was kept at one corner of the hole board for the duration of the experiment to measure its dipping its nose into a hole, which indicates curious behavior [39]. The number of head dips and the duration of each head dip (seconds) were recorded for 5 min. One head dip was considered as an animal having both eyes into a hole [40]. The testers were kept blind to the treatment procedures to minimize observational bias. Rats were tested one at a time.

#### 2.5.2. Sexual Behavior

Another set of rats was treated with the doses mentioned under experimental design. One and seven days posttreatment, the rats were placed individually in plastic boxes and were given a 10-min adaption period. Female rats were checked for their reproductive cycle stage by collecting vaginal smears. Females in estrus were paired with male rats and observations were made for 40 min. Measurements, including mount and intromission latency (the time from introduction of the female to the first mount and first intromission), ejaculatory latency (the time from the introduction of the receptive female to the first ejaculate), percent of mounts (number of mountings/number paired × 100), percent of intromission (number of intromissions/number paired x 100), percent of ejaculation (number of ejaculations/number paired × 100), copulatory efficiency (number of intromissions/number of mounts × 100), and inter-copulatory interval (average time between intromissions), were recorded. The testing was carried out during the dark period of rat cycle between 20–24 h in a separate room under faint light. If a male failed to mount 10 min after the introduction of females, it was considered as sexually inactive [4,41].

### 2.6. Fertility Test

If a rat ejaculated during the evaluation of sexual behavior, paired male and female rats stayed together for a further four hours, permitting a higher number of ejaculations. Inactive animals were tested daily for the next five days, during which different estrus females were paired. The following morning (08:00–08:30) females and males were separated and successful mating was established by the existence of spermatozoa in the vaginal smears of the females. The initial day was determined to be day zero of gestation. If sperm was observed in the smears, the spermatozoa number (×200) was estimated using an improved Neubauer hematocytometer (Fison Scientific, Loughborough, UK). If sperm was absent, the females were continuously observed for pregnancy. Return of the estrous cycle indicated nonpregnancy while continuous diestrus indicated pregnancy. On the 20th day of gestation, the females underwent laparotomies under aseptic conditions using mild ether anesthesia (isoflurane, 0.1 mL/L). Upon surgical procedure, both viable and dead uterine implants, resorption sites in each horn, and the number of corpus lutea in each ovary were recorded. Using the obtained data, fertility parameters were computed: Libido index (number mated/number paired × 100), quantal pregnancy (number of rats pregnant/number of rats mated × 100), fertility index (number pregnant/number paired × 100), implantation index (total number of implants/total number mated × 100), preimplantation loss (number of corpora lutea − number of implants/number of corpora lutea × 100) and post-implantation loss (number of implants − number of viable implants/number of implants × 100) [7]. 

Once the surgical procedure was completed, to prevent hypothermia, the animals were kept under heat lamps for 30 min and subsequently transferred to clean cages until recovery. In order to minimize pain, buprenorphine (0.1 mg/kg) was given every 12 h subcutaneously for three days.

### 2.7. Analgesic Effects

To test the analgesic effects, the procedure described by Ratnasooriya et al. [42] was employed. The animals were individually placed in the tail flick analgesia meter (model MK, 330A, Tokyo, Japan) at a beam level of 55, and the time taken (seconds) for the rat to flick its tail away from the light source (reaction time) was determined. Upon recovery, the animals were placed individually on a hot plate analgesia meter (MK 350A, Tokyo, Japan) maintained at 55 °C, and the time taken (in seconds) to lick its forepaws (reaction time) was measured. 

### 2.8. Muscle Strength and Coordination

Muscle strength and coordination were evaluated using bar and bridge tests, respectively. As described previously, the animal’s forepaw grip strength was tested to evaluate the motor power [43]. Briefly, the rat was placed, held by its tail, just above the trapeze pole, and lowered until it was able to grasp the pole with both forepaws. A steady parallel wrench was applied until the rat released its grasp. The experiment was repeated five times at 30 s intervals. If the rat forcefully jerked the pole without simply releasing its grasp, those readings were excluded. The body weight of each rat was recorded, prior to the experiment. Immediately after that, muscle coordination was examined using the bridge test. The time taken for the rat to slide off from the bridge was recorded. 

### 2.9. Blood Acetylcholinesterase Activity

Blood acetylcholinesterase (BChE) activity was measured on posttreatment days one and seven, by the method described by Antokhin et al. [44]. In the presence of BChE, the hydrolysis of Ach and production of acetic acid decreases the pH of the reaction mixture. Cholinesterase activity was expressed as (∆ pH/incubation time) = (pH1 − pH2) − ∆ pH of blank. The pH was determined using a pH meter (model pH3110.WTW Co., Weilheim, Germany). 

### 2.10. Serum Follicular-Stimulating Hormone (FSH), Luteinizing Hormone (LH), and Testosterone Levels

Upon the scarification of animals, blood was collected through heart puncture. The blood was allowed to clot at room temperature and centrifuged for 10 min to separate the serum. Serum was evaluated for concentrations of luteinizing hormone (LH), follicular-stimulating hormone (FSH), and testosterone using commercial kits (Randox Laboratories Ltd., Crumlin, UK).

### 2.11. Statistical Analysis

Statistical analysis was conducted using the Minitab statistical package (Minitab Inc, State College, PA, USA). Data were analyzed using ANOVA and Huxley’s least significant test. In the case of proportional date, G-test was used. The significance level was set to *p* ≤ 0.05.

## 3. Results

### 3.1. Behavior and External Features

No deaths or any apparent signs of toxicity, such as lacrimation or tremor, were observed among the treated or control rats. However, the rats exhibited sleepiness and slept for a longer time period following the Judo 40 treatment.

#### 3.1.1. Exploratory Behavior

The effects of Judo 40 on behavior are summarized in Table 1. At the lowest dose (20 mg/kg), all the tested behavioral parameters, locomotion (*p* < 0.01), rearing (*p* < 0.01), head dipping frequency (*p* < 0.05), and time/head dips (*p* < 0.05), were only affected significantly at posttreatment day one. At 50 mg/kg of Judo 40, a significant reduction (*p* < 0.01) in locomotion and grooming behavior was observed at both posttreatment days when compared to the control. Similar results were witnessed with the rats treated with 20 and 50 mg/kg Judo 40 when compared to the control. 

#### 3.1.2. Sexual Behavior

A significant (*p* < 0.01) decline in the percentage of mounted, intromitted, ejaculated, and percentage copulatory efficiency was observed on day one with the high dose (Table 2).

Similar to mounting, intromission and ejaculatory latencies were significantly prolonged, and the copulatory efficiency and number of intromissions until the first ejaculation were significantly (*p* < 0.01) impaired. Further, inter-copulatory interval (*p* < 0.01) was also affected. Similar results were observed with the low dose, on posttreatment day one, except for percentage mounted and number of intromissions until the first ejaculation. 

On posttreatment day seven, all the tested parameters except for percentage mounted and number of intromissions were significantly (*p* < 0.05) affected at the highest dose. However, no significant changes were observed at the low dose level except for percentage ejaculated.

### 3.2. Effects on Fertility

As summarized in Table 3, in relation to fertility, a significant (*p* < 0.01) reduction in indices of libido and fertility was observed with female rats mated with males exposed to 50 mg/kg of Judo 40, compared to the control on posttreatment days one and seven. 

Significant (*p* < 0.01) increases in pre- and post-implantation losses and reductions in the implantation index were observed only at the high dose. Both pre- and post-implantation losses and implantation indices were altered only on posttreatment day seven. Quantal pregnancy index remained unaltered at both dose levels.

### 3.3. Serum FSH, LH, and Testosterone Levels

The testosterone and LH hormone levels in the serum were reduced significantly (*p* < 0.01, *p* < 0.05) at the 50 mg/kg Judo 40 on posttreatment days one (testosterone, 42.5%; LH, 43.6%) and seven (testosterone, 19.4%; LH, 21.3%; see Table 4). However, the rats treated with 20 mg/kg Judo 40 did not display significant effects on serum hormone concentrations. 

### 3.4. Analgesic Effects

**Figure 1:**
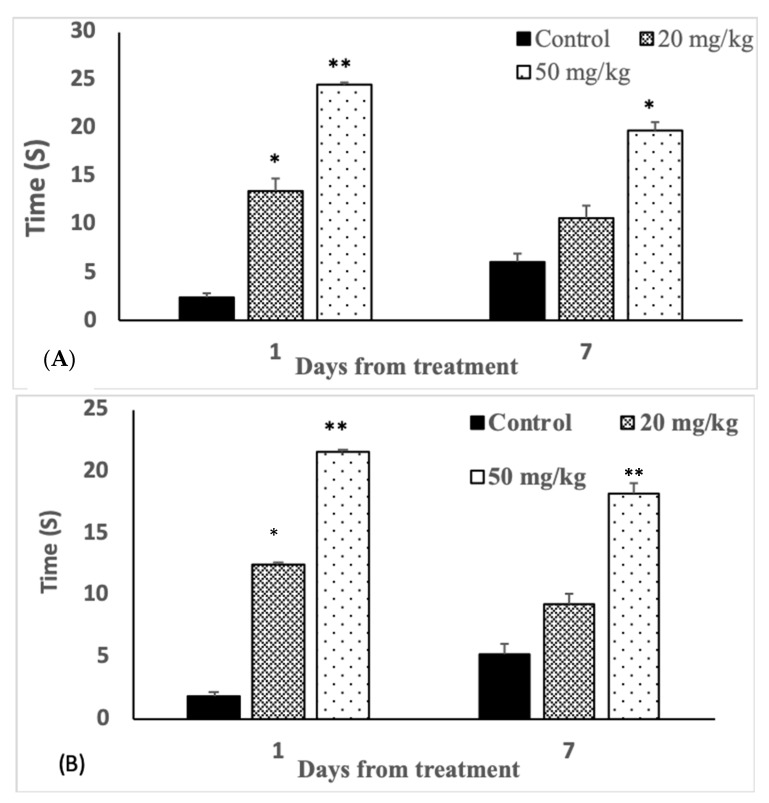
The effects of Judo 40 on the reaction time of rats (*n* = 9) treated either with Judo 40 (50 and 20 mg/kg) or the control (corn oil) as evaluated using (**A**) tail flick and (**B**) hot plate tests on the posttreatment days one and seven. The reaction time was measured in seconds. Results are expressed as mean + SEM, * *p* < 0.05, ** *p* < 0.01.

The analgesic activities of rats gavaged with 50 mg/kg Judo 40, tested using tail flick (Figure 1A) and hot plate (Figure 1B) test, exhibited a significant (*p* < 0.01) prolonged reaction time on posttreatment days one and seven. Tail flick reaction was four times and three times prolonged on days one and seven, respectively. Similarly, 20 times and three times prolonged reaction time was observed with the hot plate. However, with the lowest dose (20 mg/kg), a significant (*p* < 0.05) prolonged reaction time was observed only on the post-exposure day one with both techniques (Figure 1). 

### 3.5. Muscle Strength and Coordination

Judo 40 produced a significant (*p* < 0.01) reduction in muscle strength and coordination at the highest dose level on posttreatment day one by 44.5% (bar test) and 41.8% (bridge test), respectively. However, the effects were reversed by the posttreatment day seven (Table 5). 

### 3.6. Blood Acetylcholinesterase Level

**Figure 2:**
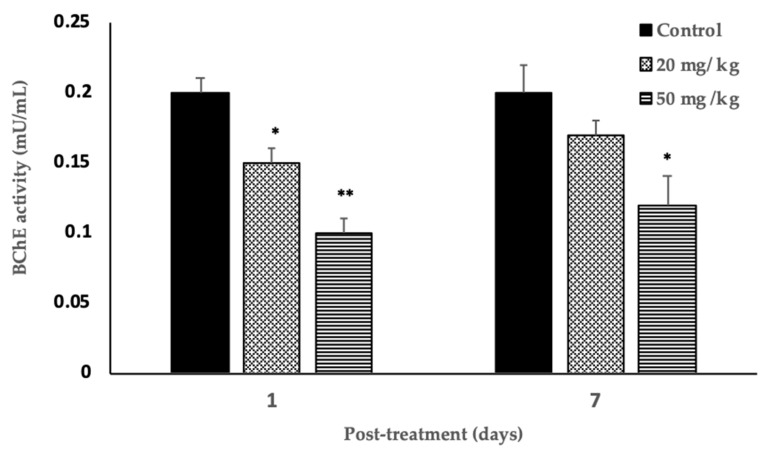
Effects of Judo 40 on BChE (blood acetylcholinesterase) activity of rats on posttreatment days one and seven. The bars represent rats (*n* = 9) treated either with Judo 40 (50 and 20 mg/kg) or the control (corn oil) on post treatment days one and seven. Results are expressed as mean ± SEM, * *p* < 0.05, ** *p* < 0.01.

Rats gavaged with both 20 and 50 mg/kg exhibited a significant decrease (*p* < 0.01) in BChE levels by 25% and 50%, respectively, compared to the control. The results are shown in Figure 2.

## 4. Discussion

The present data suggest that Judo 40 could interfere with neuronal excitability and fertility, which is evident from BChE levels, analgesic, muscle coordination, behavioral, sexual, and fertility studies. The behavioral test of open field highlights the emotional states of the animals, and it is commonly used to evaluate the chemical effects on the central nervous system.

A reduction in the plasma cholinesterase activity may not be always related to exposure to organophosphorus pesticides. On the other hand, blood acetylcholinesterase (AChE equivalent) activity is essentially influenced by organophosphates, with minor obstructions from other factors. Furthermore, the recovery of cholinesterase following organophosphate poisoning varies distinctively, with blood acetylcholinesterase (BChE) activity recovering considerably with a faster recovery of AChE activity. AChE recovery is a much slower process that takes approximately 60 to 90 days. However, the depressed BChE in mammals, including humans, is resolved with the formation of new RBC (Red Blood Cells) in the bone marrow [45]. Therefore, BChE activity can be considered a better biomarker than AChE activity [46], which was used in this study. 

Locomotor and rearing activities are indicators of exploratory behavior, and rearing is known to correlate with fear or other emotions [47]. The reduction in locomotor activities of animals treated with Judo 40 indicates that the insecticide decreased exploratory behavior [48], making animals more vulnerable in new environments. Further, the reduced rearing behavior observed in animals receiving Judo 40 suggest that the insecticide decreases emotionality. Both exploratory and rearing behaviors were inhibited significantly at both doses of Judo 50 on posttreatment day one. By posttreatment day seven, inhibition was marked only in the rats exposed to the highest dose, indicating the reversible effects at the 20 mg/kg dose level. There is an increasing concern that exposure to neuro-toxicants could alter central nervous system function, and results obtained with pain studies and muscle relaxant tests further suggest the CNS (Central Nervous System) depression effects of Judo 40. 

The recent literature has reported that a decrease in movement is one of the main extrapyramidal indicators seen among humans exposed to a broad spectrum of agricultural pesticides, and it increases with an increased exposure period [49]. The impairment of locomotion observed in the present study may partly be due to the sensory motor neuron deficit that occurs due to Judo 40 exposure. Moreover, a reduction in BChE observed in the present study may have led to impaired locomotor deficit through persistent choline action at the cholinergic receptors, leading to paralysis [42].

Results obtained from a hole-board test showed a significant decrease in the number of head dips and the time taken for a head dip in animals treated with 50 mg/kg dose of Judo 40. The hole-board provides independent information on exploration and motor activity [50]. Hence, a reduction in hole-board behavior suggests the suppressive effects of Judo 40 in the central nervous system, making animals more vulnerable when exploring new environments [42]. The reduction in locomotor activity exhibited by rats treated with Judo 40 also could lead to the suppression of exploratory behavior [49]. CPF is also known to enhance endocannabinoid signaling, thus altering the social behavior of animals [51].

Pain is a significant function of the central nervous system [52]. In the present study, Judo 40 significantly reduced the perception of pain on posttreatment day one, indicating that activities of the CNS are affected by the insecticide, through the inhibition of pain processing in the spinal cord [53]. By posttreatment day seven, the effect was reversed in animals treated with the lowest dose of Judo 40. It can also be claimed that analgesia is a result of the amalgamation of accumulation of ACh and impaired as a result of the inhibition of AChE and impaired breaking down of opioid-like compounds due to the inhibition of protease enzymes [54].

In the present study, the effects of Judo 40 on motor strength were evaluated using a bar test. The results indicated a significant decrease in forepaw motor strength, as evaluated by grip time, and the results are in agreement with previous studies, which showed a reduction in forelimb grip strength as a result of chronic exposure to chlorpyrifos, a metabolite of Judo 40 administration in rats [55]. The accumulation of ACh at the nerve junction leads to an alteration in peripheral nerve function [56], resulting in reduced grip strength [57] and loss of muscle strength [58]. The reduced grip time following Judo 40 exposure that has been recorded indicates impaired motor strength. Moreover, a reduction in motor strength can also be attributed to the alteration of muscle neural transmission, which may be due to the metabolism of ACh, resulting from AChE inhibition. Further, the recent literature highlighted the ability of chlorpyrifos to disrupt anterograde transportation in the sciatic nerve, which may also contribute to the induction of paralysis in treated groups [59]. 

The rapid breakdown of blood acetylcholine by ChE in various cholinergic neuronal paths in the central and peripheral nervous systems induces the termination of impulse transmission [8]. The enzyme inactivation, which can be induced by organophosphates, leads to a buildup of acetylcholine, resulting in the hyperactivation of nicotinic and muscarinic receptors, disrupting neural conduction. The inhibition of BChE activity observed in the treated rats could result in impaired behavioral and muscle strength and analgesic effects. Ambali and Ayo [60] reported comparable results with chlorpyrifos, the metabolite of Judo 40. On day one posttreatment, the highest dose produced a 50% inhibition of BChE, indicating interference with cholinergic receptors as a result of the down-regulation of a muscarinic receptor concentration in the pons/medulla of rats [61]. Furthermore, acute general toxicity symptoms observed in the present study are associated with declining BChE levels. Most organophosphates are degraded quickly by metabolic reaction and the elimination process. However, it has been demonstrated that a significant amount of pesticides remains in the body. BChE inhibition was significantly apparent in posttreatment day one, as indicated by all the tests, including bar and bridge tests. However, by post-exposure day seven, though BChE levels were not significantly affected, the behavioral and analgesic effects were not reversed at the 50 mg/kg dose level. At a 20 mg/kg dose level, the effects were reversed. Organophosphates exert their toxic effects in ways other than acetylcholinesterase inhibition. In the absence of cholinergic signs, numerous effects on behavioral changes can be produced as a result of reduced nerve transmission targeting other brain proteins, which are likely to display alterations in their activity and relationship to BChE-mediated acute toxicity [62]. 

The current results highlight that a short time of exposure to high doses can impair the sexual behavior and fertility of exposed rats. Pesticides disrupt the spermatogenesis process by triggering hormonal imbalances. The reduction in testosterone and LH observed in the present study could be a result of Judo 40 interfering with the hypothalamic–pituitary–testicular (HPT) axes. Pesticide exposure can lead to the impairment of the hypothalamic–pituitary–testicular (HPT) axes via a reduction in plasma LH, FSH, and testosterone levels [63]. However, in the present study, a reduction in FSH was not evident. Maintaining FSH levels is vital for the spermatogenesis process [64], and normal FSH levels in the Judo 40-exposed rats led to observed unaltered spermatozoa counts during initial studies [11]. Testosterone is produced by Leydig cells under the influence of luteinizing hormone (LH). According to published literature [29,65], exposure to CPF, the active ingredient of Judo 40, resulted in oxidative damages to Leydig cells, diminishing the production of desired testosterone levels [66]. 

Both doses (50 and 20 mg/kg) of Judo 40, induced stronger behavioral effects, especially suppressing mounting and coupling behaviors. These parameters are known as crucial phenomena of sexual behaviors, compared to initial behavior events such as sniffing and chasing [65]. Both testosterone and 17b-estradiol in the frontal region of the brain are vital for the full manifestation of male sexual behavior [67]. Low levels of serum testosterone may have resulted in impaired sexual behavior activity, particularly on posttreatment day seven. 

In the present study, it was also evident that Judo 40 can exert inhibitory effects on fertility. Progressive motility of spermatozoa and nuclear integrity is extremely important for successful fertilization [67]. Increased head abnormalities [68,69] and impaired sperm motility [70] leads to a reduction in fertilizing ability [71,72]. Exposure to Judo 40 led to impaired sperm motility and nuclear integrity [11]. Hence, the fertilization capabilities of sperm in the present study were significantly reduced because of spermatozoa movement dysfunction and a decrease in sperm DNA integrity, and this is evident from the reduction in pregnancy indices [71].

Numerous studies have given an insight into farmers with impaired fertility, linked with poor semen quality and low testosterone levels [72]. However, attention should be drawn to the fact that both men and women of reproductive age are continuously exposed to organophosphates [73], resulting in fetal abnormalities and impairment growths [74,75]. The results of the present study also confirm that continuous exposure to chemical formulations of CPF are a major concern for human health. In Sri Lanka, the occupational exposure to CPF of the farmers ranged from 2500 to 90,000 ng/kg/day [76]. Therefore, the long-term exposure to chemical formulation of CPF should raise a concern. 

In conclusion, our results demonstrate that exposure to Judo 40, the commercial formulation, induces toxic insult to the central nervous system and male reproductive system leading to impaired behavior, fertilizing ability, and hormonal imbalances. The findings have important repercussions for reproductive and fertility risk assessments. It is important to create awareness among pesticide users to wear appropriate gear to protect the farmers from exposure to this drift-prone pesticide, which is found in significant quantities as residue in sediments, crops, and water. The study also reveals that the exposure level of farmers to Judo 40 should be less than 20 mg/kg.

## Figures and Tables

**Table 1:** Effects of Judo 40 and corn oil (control) on exploratory behavioral parameters of male rats.

Parameters	Post Treatment Day 1			Post Treatment Day 7		
	Control	20 mg/kg	50 mg/kg	Control	20 mg/kg	50 mg/kg
Locomotion	16.0 ± 1.8	3.8 ± 1.5 **	5.8 ± 1.69 **	21.2 ± 2.7	19.6 ± 2.5	8.0 ± 2.56 **
Rearing	14.5 ± 3.1	6.2 ± 1.58 **	2.1 ± 0.72 **	7.5 ± 8.22	8.22 ± 1.39	3.4 ± 1.18 *
Frequency of head dipping	7.7 ± 1.34	3.6 ± 1.32 *	2.0 ± 0.77 **	11.6 ± 1.0	6.6 ± 0.08	3.5 ± 1.23 **
Time/head dip (s)	1.2 ± 0.36	2.1 ± 0.17 *	2.5 ± 0.13 *	1.9 ± 0.31	1.7 ± 0.21	2.3 ± 0.40 *

Data are presented as mean ± SEM (*n* = 6). Significantly different mean values in a column superscripted by * *p* < 0.05, ** *p* < 0.01.

**Table 2:** Sexual behavior of male rats treated either with Judo 40 or corn oil (control).

Behavioral Indices	Post-Treatment Day 1			Post-Treatment Day 7		
	Control	20 mg/kg	50 mg/kg	Control	20 mg/kg	50 mg/kg
% Mounted	100	80	18 *	100	84	66
% Intromitted	100	54 *	18 **	100	79	42 *
% Ejaculated	100	45 *	23 **	100	58 *	38 *
% Copulatory efficiency	85	60 *	19 **	90	68	40 *
No: of intromissions until the first ejaculation	17	13.5	4 **	18.5	14	11
Mounting latency (S)	44	300 **	750 **	52	250	650 *
Intromission latency (S)	32	400 **	700 **	30	38	700 *
Ejaculatory latency (S)	205	600 *	950 **	210	410	850 *
Inter-copulatory interval	74	420 **	760 **	64	102	418 *

Data are presented as mean ± SEM (*n* = 9), * *p* < 0.05, ** *p* < 0.01.

**Table 3:** Fertility indices of female rats mated with male rats treated either with 20 or 50 mg/kg Judo 40 or distilled water (control).

Fertility Indices	Post-Treatment Day 1			Post-Treatment Day 7		
	Control	20 mg/kg	50 mg/kg	Control	20 mg/kg	50 mg/kg
Libido index	100	66.7	33.5 **	100	77.8	55.3 *
Fertility index	100	77.7	22.2 **	100	75.5	35.2 *
Pre-implantation loss	22.2 ± 4.3	20.2 ± 4.2	45.1 ± 3.8 *	20.6 ± 6.2	11.8 ± 5.3	48.5 ± 8.3 *
Post implantation loss	20.6 ± 6.2	16.2 ± 9.2	18.1 ± 6.8	3.02 ± 0.8	12.6 ± 6.3	9.9 ± 4.5 *
Implantation index	11.7 ± 0.5	11.6 ± 0.7	10.0 ± 1.7	12.2 ± 0.7	11.2 ± 0.8	10.3 ± 1.6

Data are presented as mean ± SEM (*n* = 9). Significantly different mean values in a column superscripted by * *p* < 0.05, ** *p* < 0.01.

**Table 4:** Serum testosterone, follicular-stimulating hormone (FSH), and luteinizing hormone (LH) of male rats, treated either with 20 or 50 mg/kg of Judo or distilled water.

Treatment	Post Treatment Day 1			Post Treatment Day 7		
	Control	20 mg/kg	50 mg/kg	Control	20 mg/kg	50 mg/kg
Testosterone	4.0 ± 0.74	3.6 ± 0.09	2.3 ± 0.55 **	3.6 ± 0.14	4.4 ± 0.04	2.9 ± 0.15 *
FSH	2.76 ± 0.63	3.7 ± 0.07	2.3 ± 0.45 **	3.4 ± 0.12	4.0 ± 0.03	3.0 ± 0.15
LH	3.9 ± 0.71	3.2 ± 0.08	2.2 ± 0.32 **	4.7 ± 0.64	4.5 ± 0.08	3.7 ± 0.25 *

Data are presented as mean ± SEM (*n* = 9). Significantly different mean values in a column superscripted by * *p* < 0.05, ** *p* < 0.01.

**Table 5:** Effects of Judo 40 and corn oil (control) on muscle strength and coordination of male rats.

Parameters	Post Treatment Day 1			Post Treatment Day 7		
	Control	20 mg/kg	50 mg/kg	Control	20 mg/kg	50 mg/kg
Bar test (seconds)	40.0 ± 5.74	36.7 ± 7.09	23.3 ± 6.55 **	36.6 ± 4.14	44.0 ± 3.04	34.0 ± 4.15
Bridge test (seconds)	39.8 ± 1.71	37.2 ± 4.8	22.0 ± 4.2 **	47.8 ± 3.64	45.7 ± 4.8	35.7 ± 3.25 *

Data are presented as mean ± SEM (*n* = 9). Significantly different mean values in a column superscripted by * *p* < 0.05, ** *p* < 0.01.

## Appendix A

**Table A1:** Mortality data of the male rats in the determination of LD_50_.

Dose (mg/Kg)	No: Rats Treated	No: Died
Control	6	0
2	6	0
20	6	0
100	6	6
200	6	6

## References

[1] Sankoh A.I., Whittle R., Semple K.T., Jones K.C., Sweetman A.J. (2016). An assessment of the impacts of pesticide use on the environment and health of rice farmers in Sierra Leone. Environ. Int..

[2] Sharma Y., Bashir S., Irshad M., Gupta S.D., Dogra T.D. (2005). Effects of acute dimethoate administration on antioxidant status of liver and brain of experimental rats. Toxicology.

[3] Peiris L.D.C., Jayathunga Y.N.A., Ratnasooriya W.D. (1995). Antireproductive effects in male rats exposed to methamidophos. Ceylon J. Sci. Biol. Sci..

[4] Slotkin T.A., Seidler F.J. (2012). Developmental neurotoxicity of organophosphates targets cell cycle and apoptosis, revealed by transcriptional profiles in vivo and in vitro. Neurotoxicol. Teratol..

[5] Delfino R.T., Ribeiro T.S., Figueroa-Villar J.D. (2009). Organophosphorus compounds as chemical warfare agents: A review. J. Braz. Chem. Soc..

[6] Sengupta P., Borges E., Dutta S., Krajewska-Kulak E. (2018). Decline in sperm count in European men during the past 50 years. Hum. Exp. Toxicol..

[7] Ratnasooriya W.D., Jayathunga Y.N.A., Peiris L.D.C. (1995). Monocrotophos impairs the fertility of male rats. Med. Sci. Res..

[8] Colović M.B., Krstić D.Z., Lazarević-Pašti T.D., Bondžić A.M., Vasić V.M. (2013). Acetylcholinesterase inhibitors: Pharmacology and toxicology. Curr. Neuropharmacol..

[9] Richardson R.J., Hein N.D., Wijeyesakere S.J., Fink J.K., Makhaeva G.F. (2013). Neuropathy target esterase (NTE): Overview and future. Chem. Biol. Interact..

[10] Sengupta P., Nwagha U., Dutta S., Krajewska-Kulak E., Izuka E. (2017). Evidence for decreasing sperm count in African population from 1965 to 2015. Afr. Health Sci..

[11] Kudavidanage E.P., Peiris D.D. (2016). Exposure of judo 40 alters DNA integrity and sperm function of rat Multidisciplinary. EPRA Intern. J. Multidiscip. Res..

[12] Institute of Policy Studies of Sri Lanka (IPS), Cultivation Ministry of Agriculture, Sri Lanka Better Water, Sustainable Agriculture and Better Lives for Sri Lanka. Paddy Cultivation. http://www.ips.lk/talkingeconomics/2016/03/22/better-water-sustainable-agriculture-and-better-lives-for-sri-lanka/.

[13] Schipani V. The Facts on Chlorpyrifos. https://www.factcheck.org/2017/04/the-facts-on-chlorpyrifos/.

[14] Menike A.M.W., Shanthini R., Kalpage C.S., Karunaratne D.G.G.P., Kankanamge A. (2012). Chlorpyrifos contamination of fresh water in a commercial vegetable cultivation area in Sri Lanka and factors affecting contamination. J. Natl. Sci. Found. Sri Lanka.

[15] Lester Y., Sabach S., Zivan O., Dubowski Y. (2017). Key environmental processes affecting the fate of the insecticide chloropyrifos applied to leaves. Chemosphere.

[16] Dores E.F.G.C., Spadotto C.A., Weber O.L.S., Dalla Villa R., Vecchiato A.B., Pinto A.A. (2016). Environmental Behavior of Chlorpyrifos and Endosulfan in a Tropical Soil in Central Brazil. J. Agric. Food Chem..

[17] Yuan Y., Chen C., Zheng C., Wang X., Yang G., Wang Q., Zhang Z. (2014). Residue of chlorpyrifos and cypermethrin in vegetables and probabilistic exposure assessment for consumers in Zhejiang Province, China. Food Control.

[18] Voorhees J.R., Rohlman D.S., Lein P.J., Pieper A.A. (2017). Neurotoxicity in Preclinical Models of Occupational Exposure to Organophosphorus Compounds. Front. Neurosci..

[19] Panuwet P., Prapamontol T., Chantara S., Thavornyuthikarn P., Montesano M., Whiteheadjr R., Barr D. (2008). Concentrations of urinary pesticide metabolites in small-scale farmers in Chiang Mai Province, Thailand. Sci. Total Environ..

[20] Phung D.T., Connell D., Miller G., Chu C. (2012). Probabilistic assessment of chlorpyrifos exposure to rice farmers in Viet Nam. J. Expo. Sci. Environ. Epidemiol..

[21] Selvarajah A., Thiruchelvam S. (2007). Factors affecting pesticide use by farmers in Vavuniya District. Trop. Agric. Res..

[22] Tian Y., Ishikawa H., Yamaguchi T., Yamauchi T., Yokoyama K. (2005). Teratogenicity and developmental toxicity of chlorpyrifos. Reprod. Toxicol..

[23] Dinham B. (2005). Prolonged exposure to some agricultural pesticides may increase the risk of lung cancer in agricultural workers. Evid. Based Healthc. Public Health.

[24] Álvarez M., du Mortier C., Sokolic T., Cirelli A.F. (2013). Studies on the Persistence of a Commercial Formulation of Chlorpyrifos on an Agricultural Soil from Provincia de Buenos Aires, República Argentina. Water Air Soil Pollut..

[25] Álvarez M., du Mortier C., Fernández Cirelli A. (2013). Behavior of Insecticide Chlorpyrifos on Soils and Sediments with Different Organic Matter Content from Provincia de Buenos Aires, República Argentina. Water Air Soil Pollut..

[26] De Silva P.M.C.S., van Gestel C.A.M. (2009). Comparative sensitivity of Eisenia andrei and Perionyx excavatus in earthworm avoidance tests using two soil types in the tropics. Chemosphere.

[27] Zafar M.I., Van Wijngaarden R.P.A., Roessink I., Van den Brink P.J. (2011). Effects of time-variable exposure regimes of the insecticide chlorpyrifos on freshwater invertebrate communities in microcosms. Environ. Toxicol. Chem..

[28] Bhatnagar A., Yadav A.S., Cheema N. (2016). Genotoxic Effects of Chlorpyrifos in Freshwater Fish Cirrhinus mrigala Using Micronucleus Assay. Adv. Biol..

[29] Solomon K.R., Giesy J.P., Kendall R.J., Best L.B., Coats J.R., Dixon K.R., Hooper M.J., Kenaga E.E., McMurry S.T. (2001). Chlorpyrifos: Ecotoxicological Risk Assessment for Birds and Mammals in Corn Agroecosystems. Hum. Ecol. Risk Assess. Int. J..

[30] Peiris D.C., Dhanushka T. (2017). Low doses of chlorpyrifos interfere with spermatogenesis of rats through reduction of sex hormones. Environ. Sci. Pollut. Res..

[31] Corrieri L., Della Seta D., Canoine V., Fusani L. (2007). Developmental exposure to xenoestrogen enhances spatial learning in male rats. Horm. Behav..

[32] Oosthuizen M.K., Scheibler A.-G., Charles Bennett N., Amrein I. (2013). Effects of Laboratory Housing on Exploratory Behaviour, Novelty Discrimination and Spatial Reference Memory in a Subterranean, Solitary Rodent, the Cape Mole-Rat (Georychus capensis). PLoS ONE.

[33] Wang H.P., Liang Y.J., Sun Y.J., Hou W.Y., Chen J.X., Long D.X., Xu M.Y., Wu Y.J. (2014). Subchronic neurotoxicity of chlorpyrifos, carbaryl, and their combination in rats. Environ. Toxicol..

[34] Zaccaroni M., Della Seta D., Farabollini F., Fusani L., Dessì-Fulgheri F. (2016). Developmental Exposure to Very Low Levels of Ethynilestradiol Affects Anxiety in a Novelty Place Preference Test of Juvenile Rats. Neurotox. Res..

[35] Dent C.L., Isles A.R., Humby T. (2014). Measuring risk-taking in mice: Balancing the risk between seeking reward and danger. Eur. J. Neurosci..

[36] Burke R.D., Todd S.W., Lumsden E., Mullins R.J., Mamczarz J., Fawcett W.P., Gullapalli R.P., Randall W.R., Pereira E.F.R., Albuquerque E.X. (2017). Developmental neurotoxicity of the organophosphorus insecticide chlorpyrifos: From clinical findings to preclinical models and potential mechanisms. J. Neurochem..

[37] Salazar-Arredondo E., Solís-Heredia M.d.J., Rojas-García E., Hernández-Ochoa I., Quintanilla-Vega B. (2008). Sperm chromatin alteration and DNA damage by methyl-parathion, chlorpyrifos and diazinon and their oxon metabolites in human spermatozoa. Reprod. Toxicol..

[38] Tatem K.S., Quinn J.L., Phadke A., Yu Q., Gordish-Dressman H., Nagaraju K. (2014). Behavioral and Locomotor Measurements Using an Open Field Activity Monitoring System for Skeletal Muscle Diseases. J. Vis. Exp..

[39] Brown G.R., Nemes C. (2008). The exploratory behaviour of rats in the hole-board apparatus: Is head-dipping a valid measure of neophilia?. Behav. Processes.

[40] Terçariol P.R.G., Godinho A.F. (2011). Behavioral effects of acute exposure to the insecticide fipronil. Pestic. Biochem. Physiol..

[41] Agmo A. (1997). Male rat sexual behavior. Brain Res. Brain Res. Protoc..

[42] Ratnasoonya W.D., Peiris L.D.C., Amarasekera A.S. (1994). Analgesic activities of Murraya koengnigii leaf extract. Med. Sci. Res..

[43] Ratnasooriya W.D., Peiris L.D.C., Jayathunga Y.N.A. (1995). Analgesic and sedative action of monchrotophos following oral administration in rats. Med. Sci. Res..

[44] Antokhin A.M., Gainullina E.T., Ryzhikov S.B., Taranchenko V.F., Yavaeva D.K. (2009). Rapid method for measurement of acetylcholinesterase activity. Bull. Exp. Biol. Med..

[45] Mason H.J. (2000). The Recovery of Plasma Cholinesterase and Erythrocyte Acetylcholinesterase Activity in Workers after Over-exposure to Dichlorvos. Occup. Med. (Chic. Ill)..

[46] Jaga K., Dharmani C. (2003). Sources of exposure to and public health implications of organophosphate pesticides. Rev. Panam. Salud Publica.

[47] Barros H.M.T.T., Tannhauser S.L., Tannhauser M.A.L.L., Tannhauser M.A.L.L. (1994). The Effects of GABAergic Drugs on Grooming Behaviour in the Open Field. Pharmacol. Toxicol..

[48] Archer J. (1973). Tests for emotionality in rats and mice: A review. Anim. Behav..

[49] Alavanja M.C.R., Hoppin J.A., Kamel F. (2004). Health Effects of Chronic Pesticide Exposure: Cancer and Neurotoxicity. Annu. Rev. Public Health.

[50] Durcan M.J., Lister R.G. (1989). Does directed exploration influence locomotor activity in a hole board test. Behav. Neural Biol..

[51] Carr R.L., Alugubelly N., de Leon K., Loyant L., Mohammed A.N., Patterson M.E., Ross M.K., Rowbotham N.E. (2020). Inhibition of fatty acid amide hydrolase by chlorpyrifos in juvenile rats results in altered exploratory and social behavior as adolescents. Neurotoxicology.

[52] Beazley-Long N., Durrant A.M., Swift M.N., Donaldson L.F. (2018). The physiological functions of central nervous system pericytes and a potential role in pain. F1000Research.

[53] Terry A.V. (2003). Repeated Exposures to Subthreshold Doses of Chlorpyrifos in Rats: Hippocampal Damage, Impaired Axonal Transport, and Deficits in Spatial Learning. J. Pharmacol. Exp. Ther..

[54] Clement J.G., Taffy Copeman H. (1984). Soman and sarin induce a long-lasting naloxone-reversible analgesia in mice. Life Sci..

[55] Steenland K., Dick R.B., Howell R.J., Chrislip D.W., Hines C.J., Reid T.M., Lehman E., Laber P., Krieg E.F., Knott C. (2000). Neurologic function among termiticide applicators exposed to chlorpyrifos. Environ. Health Perspect..

[56] Terry A.V., Gearhart D.A., Beck W.D., Truan J.N., Middlemore M.-L.M.-L., Williamson L.N., Bartlett M.G., Prendergast M.A., Sickles D.W., Buccafusco J.J. (2007). Chronic, intermittent exposure to chlorpyrifos in rats: Protracted effects on axonal transport, neurotrophin receptors, cholinergic markers, and information processing. J. Pharmacol. Exp. Ther..

[57] Steenland K., Jenkins B., Ames R.G., O’Malley M., Chrislip D., Russo J. (1994). Chronic neurological sequelae to organophosphate pesticide poisoning. Am. J. Public Health.

[58] Miranda J., McConnell R., Wesseling C., Cuadra R., Delgado E., Torres E., Keifer M., Lundberg I. (2004). Muscular strength and vibration thresholds during two years after acute poisoning with organophosphate insecticides. Occup. Environ. Med..

[59] Gao J., Naughton S.X., Wulff H., Singh V., Beck W.D., Magrane J., Thomas B., Kaidery N.A., Hernandez C.M., Terry A.V. (2016). Diisopropylfluorophosphate Impairs the Transport of Membrane-Bound Organelles in Rat Cortical Axons. J. Pharmacol. Exp. Ther..

[60] Ambali S., Ayo J. (2012). Vitamin C attenuates chronic chlorpyrifos-induced alteration of neurobehavioral parameters in Wistar rats. Toxicol. Int..

[61] Nostrandt A.C., Padilla S., Moser V.C. (1997). The Relationship of Oral Chlorpyrifos Effects on Behavior, Cholinesterase Inhibition, and Muscarinic Receptor Density in Rat. Pharmacol. Biochem. Behav..

[62] Ray D.E., Richards P.G. (2001). The potential for toxic effects of chronic, low-dose exposure to organophosphates. Toxicol. Lett..

[63] Pandey S.P., Tsutsui K., Mohanty B. (2017). Endocrine disrupting pesticides impair the neuroendocrine regulation of reproductive behaviors and secondary sexual characters of red munia (Amandava amandava). Physiol. Behav..

[64] Plant T.M., Marshall G.R. (2001). The functional significance of FSH in spermatogenesis and the control of its secretion in male primates. Endocr. Rev..

[65] Matsumoto J., Urakawa S., Hori E., de Araujo M.F.P., Sakuma Y., Ono T., Nishijo H. (2012). Neuronal Responses in the Nucleus Accumbens Shell during Sexual Behavior in Male Rats. J. Neurosci..

[66] Janssens L., Stoks R. (2017). Chlorpyrifos-induced oxidative damage is reduced under warming and predation risk: Explaining antagonistic interactions with a pesticide. Environ. Pollut..

[67] Gillies G.E., McArthur S. (2010). Estrogen actions in the brain and the basis for differential action in men and women: A case for sex-specific medicines. Pharmacol. Rev..

[68] Dhanushka M.A.T., Peiris L.D.C. (2017). Cytotoxic and Genotoxic Effects of Acephate on Human Sperm. J. Toxicol..

[69] Peiris L.D.C., Chathu P., Perera D.D.B.D., Moore H.D. (2019). 1,3-Dinitrobenze-Induced Genotoxicity Through Altering Nuclear Integrity of Diploid and Polyploidy Germ Cells. Dose-Response.

[70] Peiris L.D., Moore H.D. (2001). Evaluation of effects of 1,3-dinitrobenzene on sperm motility of hamster using computer assisted semen analysis (CASA). Asian J. Androl..

[71] Dutta A.L., Sahu C.R. (2013). Emblica officinalis Garten fruits extract ameliorates reproductive injury and oxidative testicular toxicity induced by chlorpyrifos in male rats. Springerplus.

[72] Peiris-John R.J., Wickremasinghe R. (2008). Impact of low-level exposure to organophosphates on human reproduction and survival. Trans. R. Soc. Trop. Med. Hyg..

[73] Rauh V.A., Perera F.P., Horton M.K., Whyatt R.M., Bansal R., Hao X., Liu J., Barr D.B., Slotkin T.A., Peterson B.S. (2012). Brain anomalies in children exposed prenatally to a common organophosphate pesticide. Proc. Natl. Acad. Sci. USA.

[74] Cognez N., Warembourg C., Zaros C., Metten M.-A., Bouvier G., Garlantézec R., Charles M.-A., Béranger R., Chevrier C. (2019). Residential sources of pesticide exposure during pregnancy and the risks of hypospadias and cryptorchidism: The French ELFE birth cohort. Occup. Environ. Med..

[75] von Ehrenstein O.S., Ling C., Cui X., Cockburn M., Park A.S., Yu F., Wu J., Ritz B. (2019). Prenatal and infant exposure to ambient pesticides and autism spectrum disorder in children: Population based case-control study. BMJ.

[76] Marasinghe J., Yu Q., Connell D. (2014). Assessment of Health Risk in Human Populations Due to Chlorpyrifos. Toxics.

